# Embolization of Cyanoacrylate glue in systemic circulation in a case of hepatocellular carcinoma: an autopsy report

**DOI:** 10.1186/1746-1596-4-45

**Published:** 2009-12-09

**Authors:** Kirti Gupta, Rakesh K Vasishta, Usha Dutta, Rakesh K Kochhar, Kartar Singh

**Affiliations:** 1 Department of Histopathology, Post Graduate Institute of Medical Education and Research, (PGIMER), Chandigarh, India; 2 Department of Gasteroenterology, Post Graduate Institute of Medical Education and Research, (PGIMER), Chandigarh, India

## Abstract

We report a case of embolism of the sclerosant dye with subsequent formation of foreign-body giant cell reaction within the veins of pulmonary and portal circulation in an autopsy case of hepatocellular carcinoma developing over an underlying primary biliary cirrhosis.

## Clinical Summary

A 77 year-old lady presented four years back with upper gastrointestinal bleed. On examination, she had splenomegaly and grade three esophageal and fundal varices. Ultrasound abdomen had revealed a shrunken liver with coarse echotexture, portal vein (15 mm), splenomegaly, ascites and collateral formation at gastrohepatic ligament. Viral markers [anti-Hepatitis C virus (anti-HCV) and hepatitis B surface antigen (HBsAg)] were negative. Ascitic fluid examination revealed a total cell count of 80 (all lymphocytes), protein 300 mg/dL, sugar 134 mg/dl, SAAG 2.5. She was diagnosed as a case of decompensated cirrhosis with portal hypertension, ascites, upper gastrointestinal bleed, and hepatic encephalopathy. She was discharged on oral hematinics, Propranolol, diuretics and Lactulose. She underwent several sessions of variceal ligation, endoscopic sclerotherapy for esophageal varices. She also underwent 2 sessions of cyanoacrylate glue injection (1.5 ml each time) for large fundal varices. A year later, she developed persistent pruritis. Autoimmune work-up revealed anti-nuclear antibody (ANA) 3+, other autoimmune markers [(anti-smooth muscle autoantibody (SMA), anti-liver/kidney microsome antibody (LKM) and anti-mitochondrial antibody (AMA)] were negative. The liver biopsy was deferred in view of persistent deranged coagulation profile. She was started on Azathioprine (50 mg) which had to be stopped after 9 months because of persistent elevation of liver enzymes (aspartate aminotransferase (AST) - 100 IU/L, alanine transaminase (ALT) - 56 IU/L and alkaline phosphatase (ALP) - 622 IU/L) suggestive of Azathioprine induced cholestatic hepatitis. On subsequent follow up, her AST and ALT values normalized on stoppage of the drug.

She was now hospitalized, after 4 years, with symptoms of pleuritic chest pain for 2 weeks, with associated dyspnoea and orthopnea. She also had malena for two days about a week ago. She had pallor, icterus, peripheral cyanosis and bilateral pedal edema and tachycardia. Respiratory system examination revealed left mammary and axillary bronchial breath sounds with reduced air entry. Abdominal examination revealed hepatosplenomegaly with mild ascites. The peripheral blood examination revealed anemia with neutrophilic leucocytosis. There was conjugated hyperbilirubinemia (8.0/6.9) with raised AST/ALT (803/243 IU/L) and alkaline phosphatase (141 U/L). The total serum proteins were reduced (3.7 gm/l) with hypoalbuminemia (1.6 gm/l). The coagulogram was deranged with prothrombin time index of 77%. The renal functions were deranged with raised blood urea (100 mg/dl) and serum creatinine (1.25 mg/l). The ascitic fluid revealed an exudative ascitic fluid with elevated proteins and cell count of 320, with predominantly polymorphs, serum-ascites albumin gradient (SAAG) of 2.1, and adenosine deaminase (ADA) level of 3.0. The chest radiograph showed left lower zone consolidation. The patient was started with injectable antibiotics (Ceftriaxone), Levofloxacin and Terlipressin, Lactulose and Metronidazole. The patient developed refractory shock and succumbed to her illness. Clinically a diagnosis of cirrhosis with portal hypertension (autoimmune liver disease) was suspected and the cause of death being community acquired severe left lobar pneumonia.

## Autopsy findings

**Figure 1:**
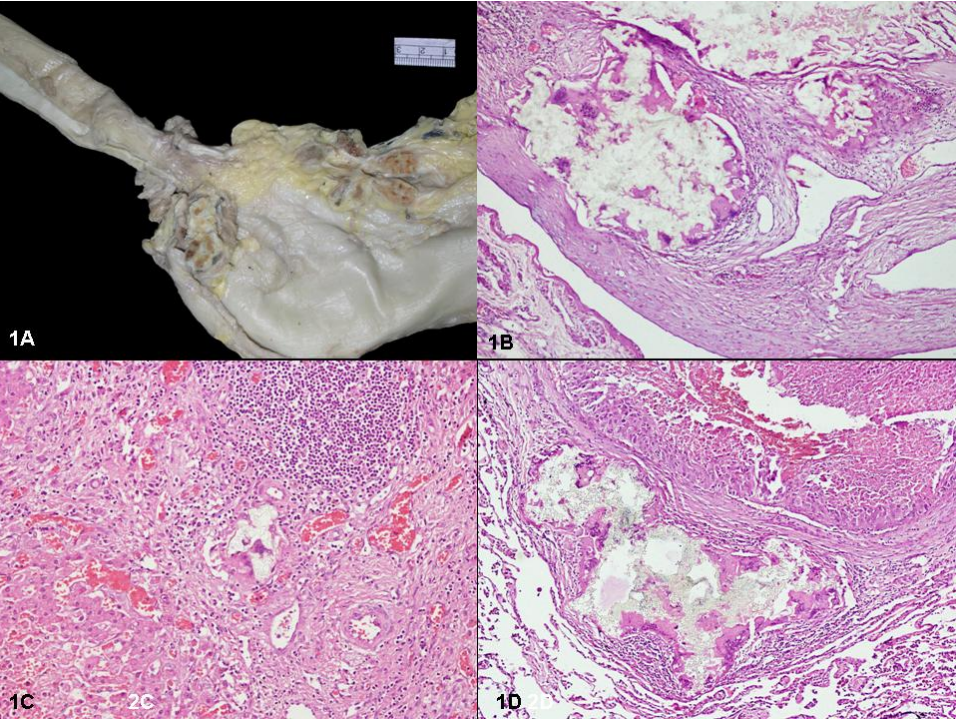
**A: Calcified veins at the gastro-oesophageal junction and along the lesser curvature**. B: Prominent giant cell reaction surrounding dye material within the vessels wall. (H&E × 100). C: Giant cell reaction around sclerosant dye within the terminal branches of portal vein (H&E × 200). D: Collection of giant cells around the dye within the wall of pulmonary vein which also shows tumor emboli within its lumen (H&E × 100).

**Figure 2:**
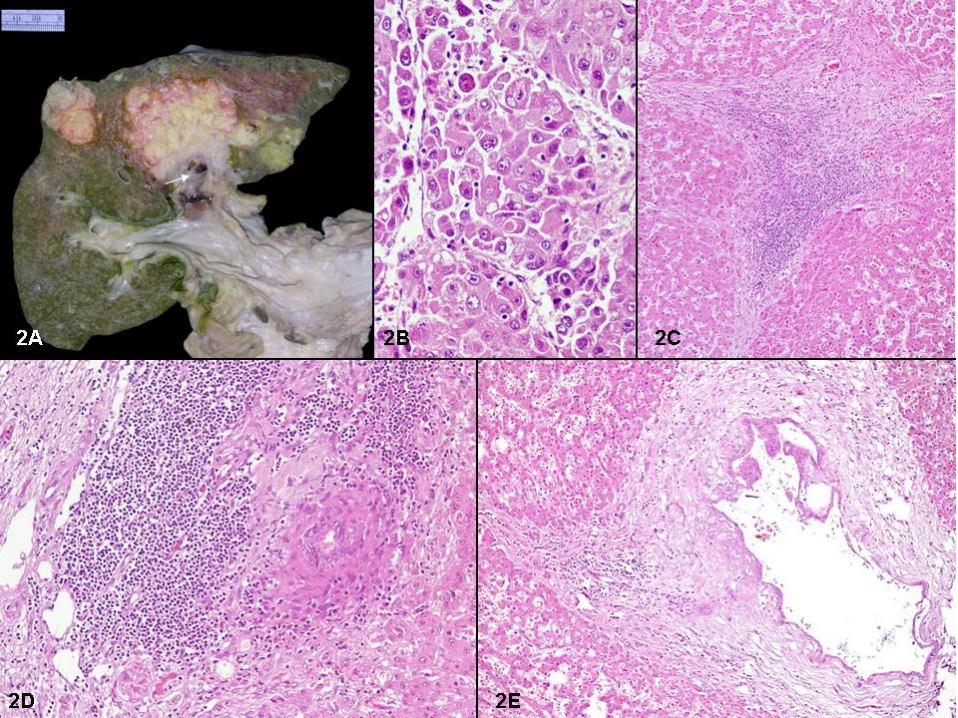
**A: Liver with multiple nodules in the left lobe, largest (5 × 6 cm) with thrombus in the left main branch of the portal vein (arrow)**. B: Neoplastic cells with prominent nucleoli, abundant cytoplasm and some with hyaline globules within it (arrow) (H&E × 400). C: Portal triad with dense lymphocytic infiltrate. Note the absence of bile ducts. (H&E × 40). D: Dense lymphocytic infiltrate present beside damaged bile duct with surrounding epithelioid granulomatous reaction (H&E × 100). E: Larger interlobular bile ducts with hyperplastic changes in the lining epithelium (H&E × 40).

A partial autopsy was performed after an informed consent. The patient was found to be icteric with haemorrhagic ascites (3 L), bilateral pleural (700 ml) and pericardial effusion (100 ml). The lower end of oesophagus showed a stricture with prominent calcified and sclerosed veins (0.8-1 cm D) at gastro-oesophageal junction, and along the lesser curvature (fig 1A). Microscopy confirmed the presence of oesophageal varices with varying degree of intimal fibrosis and proliferation of the submucosal veins. Additionally, a prominent giant cell reaction was noted in the lumen of the veins with collection of many multinucleated giant cells around the crystalline dye, at places this had eroded into the vessel wall (fig 1B). A similar type of giant cell reaction around the sclerosant dye was also noted focally in one of the branches of portal vein (fig 1C). Few pulmonary veins also revealed similar sclerosant dye within its wall with surrounding giant cell reaction (fig 1D). The liver weighed 1100 gms with multiple well-defined varying sized nodules (1-5.5 cm D) both in right and left lobes (segments II, III, IVa, IVb, VIII) (fig 2A). The rest of the liver parenchyma showed fine small nodules (1-3 mm D) conforming to the monolobar type of cirrhosis. A thrombus was identified in the left main intrahepatic branch of portal vein (fig 2A). The extrahepatic biliary system was within normal limits. Microscopically, the well defined nodules revealed a hepatocellular carcinoma with cells arranged in trabecular pattern, having prominent nucleoli, presence of hyaline globules, bi-and multinucleation and many multipolar mitotic figures (fig 2B). The neoplastic cells revealed cytoplasmic positivity with Hep-Par2 and alpha-fetoprotein antibody. Electron microscopy revealed presence of parallel and clumped arrays of intermediate filaments (conforming to Mallory bodies). The non-neoplastic liver parenchyma showed destruction of normal interlobular bile ducts within the portal tracts, few showed presence of lymphocytes within the biliary epithelium with formation of lymphoid aggregates (fig 2C). Few of the portal triads also showed fibrosis and presence of occasional epithelioid cell granulomas (fig 2D). The larger bile ducts showed hyperplastic changes with small papillae formation of the lining and vacuolation of the cytoplasm (fig 1E). The rest of this non-neoplastic liver parenchyma showed monolobar cirrhosis with formation of micronodules and porto-portal bridging fibrosis. No bridging necrosis was identified. The hepatocytes showed canalicular and cytoplasmic cholestasis, abundant Mallory hyaline bodies and increase in copper associated protein. There was no lobular inflammation and no significant interface hepatitis. Immunostaining for Hepatitis B virus surface and core antigen were negative. There was fibrocongestive splenomegaly with spleen weighing 290 gms. The lungs (980 gms) were subcrepitant and revealed fibrinous pleuritis. Multiple nodules (2-3 cm) were present bilaterally on the pleural surface. The left main pulmonary artery showed a tumor thrombus with lower lobe consolidation. Microscopically, metastatic tumor was identified in the nodules, with lympho-vascular emboli and tumoral pneumonia. In addition, there was confluent bronchopneumonia with pulmonary thromboembolism and infarcts. Grade I pulmonary arterial hypertension was noted with intimal sclerosis in the pulmonary veins. Metastatic tumor deposits were noted in the hilar lymph nodes. The kidneys (310 gms) showed features of benign arterionephrosclerosis. No features of secondary glomerulonephritis were noted. The left circumflex artery showed 50-60% atherosclerotic narrowing of its lumen and an old healed myocardial infarct was identified in the lateral wall of the left ventricle. A small tumor deposit was also noted microscopically lodged in the right ventricular wall. The bone marrow was normocellular for age but revealed marked osteoporotic changes in the bony trabeculae. The entire gastrointestinal tract was within normal limits.

## Discussion

Bleeding gastric varices are increasingly being obliterated with the aid of endoscopic injection of n-butyl-cyanoacrylate (histoacryl). This glue acts as a tissue adhesive that polymerizes on contact with blood in a gastric varix [1]. Despite the fact that cyanoacrylates, a group of rapidly polymerizing adhesives, are used widely in general surgery, neuroradiology, otolaryngology, and plastic surgery, scientific data on histopathological changes resulting from the deposition of n-butyl-2-cyanoacrylate (NBCA), a new-generation cyanoacrylate derivative, in human tissues is based largely on experimental observations in animals and sporadic postmortem studies in humans[2].

This autopsy case describes the rare occurrence of embolization and deposition of dye material into the vessel wall of systemic circulation 4 years after sclerotherapy and eliciting a foreign-body giant cell reaction. Additionally, presence of an underlying hepatocellular carcinoma which developed on a cirrhotic liver secondary to primary biliary cirrhosis/autoimmune cholangiopathy is also of rare occurrence.

Experimental studies have documented that after the injection of sclerosant dye, the main manifestation of histopathology at 3 days to 2 weeks is an acute inflammatory reaction which progresses to subacute vasculitis at 3 weeks and a chronic granulomatous foreign body reaction developing at 4 weeks. The glue mass essentially disappears in 2-3 months, and replaced by fibrotic tissue with partial vascular recanalization. At 3 weeks after injection, the elastic fibrils of the arterial wall proliferate distinctly, resulting in narrowing of the lumen with subsequent obliteration, whereas the venous wall still shows inflammation and necrosis without hyperplasia of elastic fibrils. Extrusion of glue is observed over 1-3 months in both arteries and veins and is more obvious in the latter. The histopathological changes after injection of N-butyl-2-cyanoacrylate were similar in the arteries and the veins with the exception of hyperplasia of elastic fibrils in the arterial wall and inflammation and necrosis in the venous wall at 2-3 weeks. Glue extrusion is seen in both arteries and veins [3].

Marked foreign body granulomatous response with giant cell formation, and later extreme thinning of the vessel wall, splitting of the elastic lamina, and calcification of the media have been reported in a study done to examine the effects of the butylcyanoacrylate adhesives (Histoacryl) on vascular tissue [3]. However, such kind of foreign-body giant cell reaction within the vessel wall to the dye at a distant site (pulmonary and portal circulation) secondary to embolization as seen in the present case has not been described in the literature so far. The extensive presence of dye material with giant cell reaction within the walls of larger pulmonary veins and smaller radicals of portal veins is indicative of its embolization and spread through the systemic circulation. The persistence of sclerosant dye within the vessel wall four years after sclerotherapy is of rare occurrence.

Primary biliary cirrhosis (PBC) is a chronic autoimmune liver disease [4], and several reports have shown that it is a risk factor in development of hepatobiliary malignancies [5]. The AMA negative subgroup of PBC recently termed as 'autoimmune cholangitis', is similar clinically, biochemically and histologically to PBC. Both are ANA reactive. The liver in the present case also revealed destruction of bile ducts by lymphocytes, fibrosis and occasional presence of granulomas in the portal tracts and variable degree of proliferation and hyperplastic changes in the surviving interlobular bile ducts. Cirrhosis is a risk factor for HCC development. In a patient of PBC with cirrhosis, no PBC-specific risk factors other than cirrhosis per se are present for the development of HCC [6]. However some studies state that in PBC, besides cirrhosis, male gender, hepatitis C virus (HCV) superinfection, and history of blood transfusion are associated with the development of HCC [7]. In the present case, there was no past history of blood transfusion and the viral markers were negative.

In conclusion, the present case highlights the rare occurrence of dissemination of sclerosant dye to systemic circulation and later incorporation of the dye material within the vessel wall at a distant site years after variceal ligation. Additionally, the presence of underlying primary biliary cirrhosis together with HCC developing over it lends more rarity to the present case.

## Competing interests

The authors declare that they have no competing interests.

## Authors' contributions

KG, analysed the clinical summary, worked up the autopsy and wrote the manuscript and prepared the final draft for submission. RKV, did the critical revision and worked up the autopsy case. UD, RK, KS, treated the patient and provided the clinical summary. All authors read and approved the final manuscript

## Consent

Written informed consent was obtained from the patient's relative for publication of this case report and accompanying images. A copy of the written consent is available for review by the Editor-in-Chief of this journal
